# Optimal Time of Thermotherapy for Reducing Pain, Anxiety, and Side Effects in Arteriovenous Fistula Puncture Patients: A Randomized Controlled Trial

**DOI:** 10.3390/ijerph17197147

**Published:** 2020-09-29

**Authors:** Yangok Back, Yoonyoung Lee

**Affiliations:** 1 Department of Nursing, Yeosu Hankook Hospital, 10 Yeocheoncheyukgongwon-gil, Yeosu, Jeonnam 59684, Korea; 21kiknai@naver.com; 2 Department of Nursing, Sunchon National University, 255 Jungang-ro, Suncheon, Jeonnam 57922, Korea

**Keywords:** arteriovenous fistula, puncture, thermotherapy, pain, anxiety, randomized controlled trial

## Abstract

Chronic renal failure patients undergoing hemodialysis complain of moderate pain from repeated punctures of the arteriovenous fistula. This study examined the optimal application time of thermotherapy for reducing pain, anxiety, and side effects during arteriovenous fistula puncture. This study was conducted as a single-blinded randomized controlled trial. The participants were arteriovenous fistula puncture patients with chronic renal failure who were divided into two thermotherapy groups and one control group. This study was approved by the institutional review board and registered with the Clinical Research Information Service (KCT0003768). Differences between groups regarding pain, anxiety, and side effects were analyzed using one-way ANOVA, the χ^2^ test, and the Scheffé test. A significant difference was observed between the 10-min and 20-min thermotherapy groups and the control group in terms of the pain they experienced. Additionally, more side effects were encountered in the 20-min thermotherapy group than in the 10-min group. The 10-min application of thermotherapy for an arteriovenous fistula puncture showed the same pain-reducing effect as the conventional 20-min application. The study confirmed a 10-min application of thermotherapy to be an effective nursing intervention for pain relief without side effects.

## 1. Introduction

Patients with chronic renal failure have to undergo lifelong hemodialysis. Every time the procedure is performed, an arteriovenous puncture is conducted with a 15–16-gauge thick needle. Notably, 47% of hemodialysis patients are afraid of needles and stressed. Furthermore, if the blood flow rate falls during hemodialysis, attempts to re-puncture cause more skin damage, pain, anxiety, and fear [1]. Hemodialysis patients experience anxiety before needle insertion due to repeated punctures, and over 90% of patients experience acute pain during the process [2]. 

Hemodialysis patients complained of moderate pain at four to five points of punctures in the arteriovenous fistula [3,4]. As effective drugs for reducing pain, a 2% lidocaine intradermal injection, a 10% lidocaine spray, local anesthetic cream, and a lidocaine patch are used in various ways [5,6,7]. This pharmacological intervention is effective in reducing pain during puncture of the arteriovenous fistula, but it adds cost, and it is difficult to apply a proper amount of the drug. Moreover, it is difficult to apply to hemodialysis patients in the long term because of side effects on the skin due to its prolonged use [5,6]. Furthermore, confirming the treatment time and reaction time of drug applications increases the nurse’s workload [5,6,7]. In addition to various pharmacological interventions, non-pharmacological interventions, such as cold/heat therapy, aroma massages, and interest conversion therapy, have been studied and found to be effective in reducing pain [3,8,9]. It has been reported that warm pack therapy is easier than the application of topical anesthetic cream, depending on the preferences and economic conditions of the subjects [10]. 

Thermotherapy, which is a non-pharmacological intervention method, promotes metabolism by smooth muscle relaxation and blood circulation with physiological warming effects, and reduces pain by alleviating the congestion of deep tissue [11,12]. In particular, using a hot pack has a physiological warming effect on the body due to conduction and radiation and stimulates the skin and basic tissues to reduce pain, muscle spasms, or inflammation [13]. The therapeutic effect appears when the skin tissue temperature rises from 40 to 45 °C for about 5 to 30 min [13]. When the skin temperature rises to 40 °C, the blood flow increases 4.5 times, and if the heat transfer continues for more than 30 min, the blood flow reaches the highest level and gradually decreases.

Thereafter, congestion does not persist, even if the heat transfer time is increased [14]. Lehmann and Delateur [15] stated that the thermal application time was at least 5 min for obtaining a tissue temperature response, and the safe treatment duration was 20 to 30 min, at which a maximum response was obtained at 44 °C. In other studies, thermotherapy was found to be effective when applied for about 20 to 30 min at 40 to 45 °C. If heat is continuously applied for more than 30 min, the application site exhibits tissue vasoconstriction and congestion by increasing the permeability of the capillaries [16,17,18]. 

In a previous study, thermotherapy was found to be effective in reducing pain during puncture of the arteriovenous fistula when the heating pad was heated to 48 °C and applied for 20 min [3]. In another study, it was applied to several subjects, such as breast cancer, degenerative arthritis, and percutaneous coronary intervention patients, and was effective in reducing pain in all cases [19,20,21]. In earlier studies [3,19,20], thermotherapy was applied to various subjects, but the ideal application time could not be confirmed because the application time was different. In addition, most hemodialysis patients have anxiety due to pain from arteriovenous fistula puncture [22]. Thermotherapy is known to reduce the patient’s anxiety and provide comfort [19,23], but it is unknown whether the effect is related to the response to thermotherapy. Previous studies have confirmed that thermotherapy reduces pain during arteriovenous fistula puncture, but the limitation of these studies was that they were not randomized controlled trials or studies on the application time and side effects of thermotherapy [3,10,12]. The purpose of this study is to check the optimal application time of thermotherapy for reducing pain, anxiety, and side effects during puncture of the arteriovenous fistula.

## 2. Materials and Method

### 2.1. Study Design

This study was conducted as a single-blind randomized controlled trial.

### 2.2. Participants

The subjects of this study were patients with end-stage renal failure who regularly undergo hemodialysis in hospitals in the province of South Korea. Inclusion criteria were as follows: Those who understood the purpose of this study and agreed to participate in the study; those were able to communicate; normal skin at the puncture site; and a hemodialysis period of one month or more. Exclusion criteria were as follows: Complications associated with the arteriovenous fistula; hypersensitivity reactions and circulatory disorders due to thermotherapy; and experience of applying thermotherapy to the arteriovenous puncture site. 

The sample size calculation was based on the effect size of a previous study [23,24] applying thermotherapy and varied from 0.25 to 0.8. In this study, in order to compare the differences between groups according to the formula of Cohen [25], an effect size of 0.8 and a significance level of 0.05 were calculated. As a result, the sample size required 63 patients, and a total of 80 people were recruited considering the dropout rate of 20%. 

### 2.3. Randomization and Blinding

Patient randomization was performed using a computer-generated randomized list. Randomization was conducted at a 1:1:1 ratio between groups and stratified in four patients at a time. After the researcher sealed the envelope in a randomly assigned sequence, the research assistant opened the envelope before the intervention was applied to the patient. Research assistant A applied the intervention, and research assistant B measured the effectiveness of the intervention. 

### 2.4. Intervention

Data collection was conducted from June 1 to 30, 2019, at a hospital in Y city, South Korea. In the study, experimental group 1 was 10-min thermotherapy, experimental group 2 was 20-min thermotherapy, and the control group was standard nursing care without thermotherapy. 

**Figure 1:**
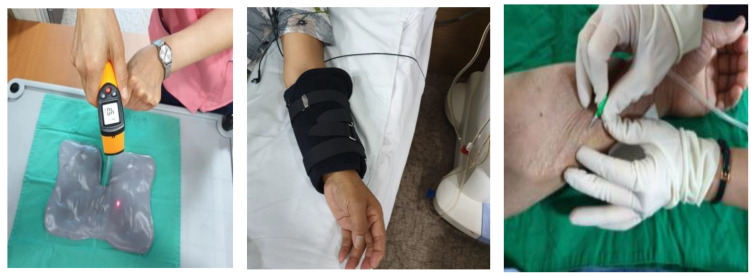
Procedure of thermotherapy.

The application device was a 500 g hot pack containing a silicate gel of 32 × 25 cm that could cover the puncture site of the arteriovenous fistula. An appropriate and safe temperature for the application of thermotherapy to be performed before puncturing the arteriovenous fistula is 40 °C, according to a previous study [3,16]. The hot pack was heated in the microwave for 2 min and then applied after confirming that its temperature had reached 40 °C with an infrared instrument (GM-700, Shenzhen, China). Arteriovenous fistula puncture was performed after the intervention assigned to the group (Figure 1).

### 2.5. Outcome

**Figure 2:**
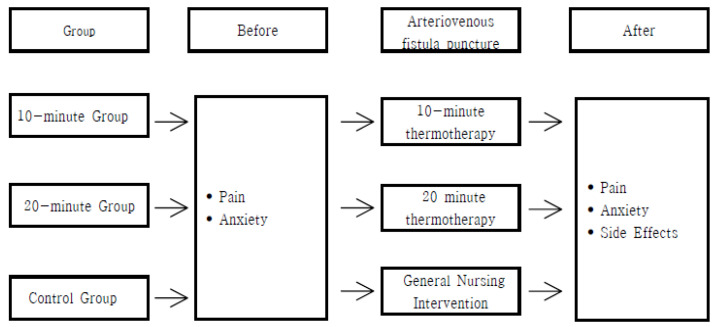
Flow chart for the time points of pain, anxiety, and adverse effects.

Pain and anxiety were measured using a visual analog scale (VAS). Research assistant B confirmed whether adverse effects such as redness, irritation, edema, and blisters occur when applying thermotherapy. Figure 2 shows a flow chart for the time points of pain assessments, anxiety assessments, and adverse effects assessments.

### 2.6. Ethical Considerations

All subjects gave prior informed consent for inclusion in the study. The study was conducted in accordance with the Declaration of Helsinki, and the protocol was approved by the institutional review board (IRB) (1040173 -201,905 -HR-015-02) at the Bioethics Review Committee of the institute affiliated with the researcher and registered with the Clinical Research Information Service (KCT0004264). 

### 2.7. Data Analysis

The data were analyzed following the per-protocol principle. Using SPSS 23.0 (IBM Corp., Armonk, NY, USA), general participant characteristics were analyzed using descriptive statistics. The homogeneity test for the three groups was analyzed using the χ^2^ test and one-way ANOVA. The comparison of pain, anxiety, and side effects of the three groups was analyzed by one-way ANOVA, and the post-test was analyzed by the Scheffé test.

## 3. Results

### 3.1. Characteristics of Subjects and Homogeneity

**Figure 3:**
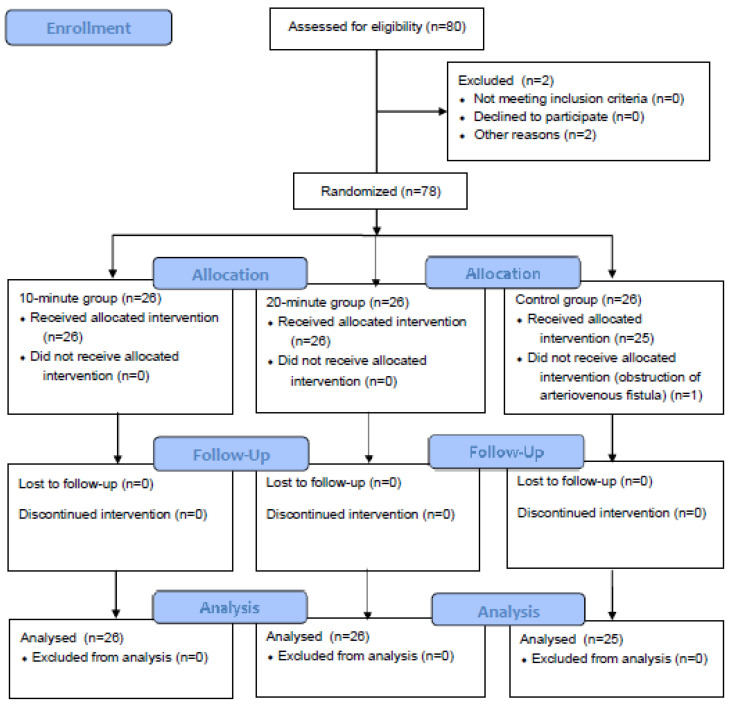
CONSORT flow diagram.

Figure 3 shows the CONSORT flow diagram. Of the 80 selected study subjects, one was transferred to another hospital due to complications (*n* = 1) and one died due to dyspnea (*n* = 1), and the remaining 78 patients were randomly assigned to the experimental group and the control group. After randomization, one patient was definitively excluded from the control group due to arteriovenous fistula obstruction. 

Table 1 shows the general characteristics and homogeneity of the three groups. There were no statistically significant differences between the three groups in terms of the general characteristics, anxiety before providing intervention, and pain at past punctures. 

### 3.2. Difference in Pain Experienced by the Three Groups According to the Application Time of Thermotherapy

**Figure 4:**
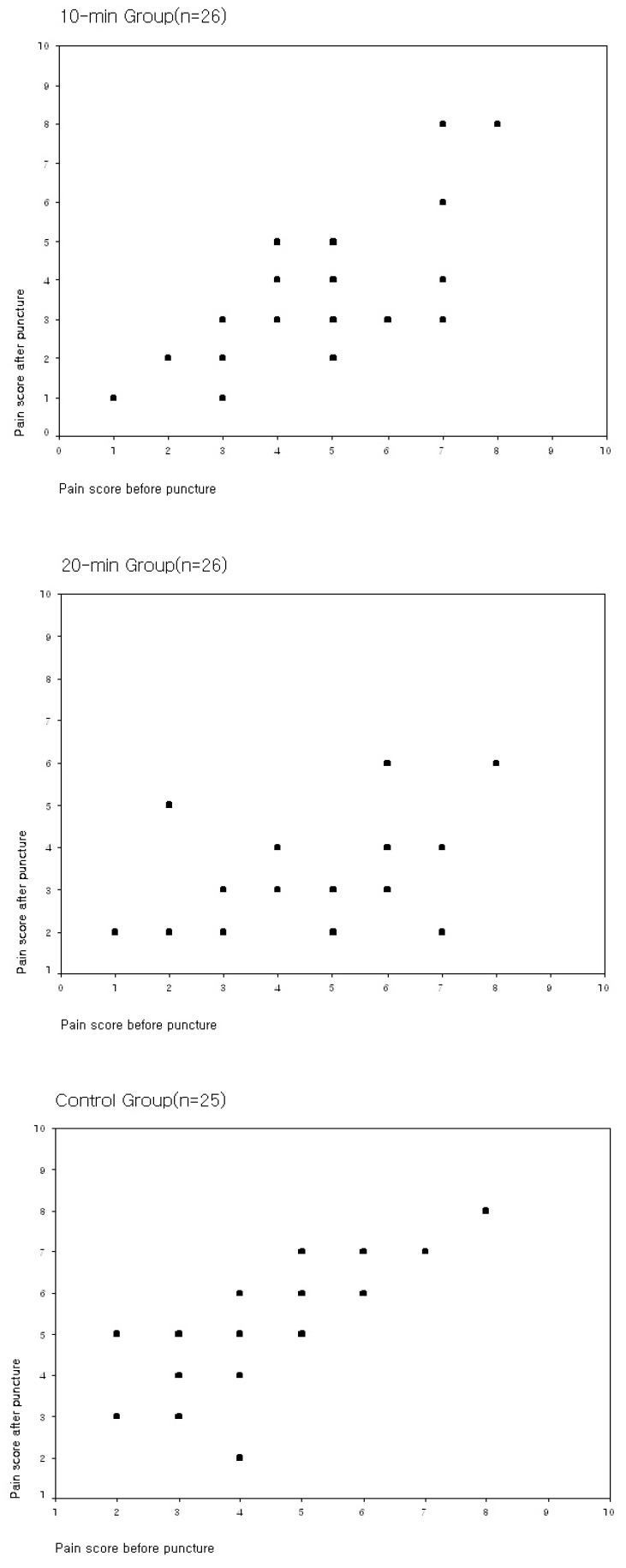
Scatter plot of the pain scores before and after puncture of the three groups.

Experimental group 1’s pain scores (3.34 ± 1.83) and experimental group 2’s pain scores (3.19 ± 1.13) were significantly lower than those of the control group (5.08 ± 1.63). (F = 11.5, *p* < 0.001) (Table 2). Figure 4 shows a scatter dot plot of the pain scores before and after arteriovenous fistula puncture of the three groups.

### 3.3. Difference in Anxiety Felt by the Three Groups According to the Application Time of Thermotherapy

There were no statistically significant differences in anxiety scores between the three groups. However, the two experimental groups had less anxiety than before the thermotherapy, but this factor had increased in the control group (Table 2). 

### 3.4. Difference in Adverse Events of the Three Groups According to the Application Time of Thermotherapy

There were no adverse events in the two experimental group for redness, edema, blisters, and pain. However, four patients (15%) in experimental group 2 complained of pruritus (Table 3).

## 4. Discussion

This study was conducted to confirm pain, anxiety, and side effects according to the duration of application of thermotherapy during puncture of the arteriovenous fistula in hemodialysis patients. 

The study found that the groups that underwent thermotherapy for 10 and 20 min experienced a pain reduction effect compared to the control group. These results are consistent with the results of previous studies showing that the pain score of the group to which the thermotherapy was applied at the time of puncture of the arteriovenous fistula was lower than that of the control group without any treatment [3,5]. This study was the result of a randomized control experiment design that was able to scientifically confirm the effect of thermotherapy on pain during puncture of the arteriovenous fistula. 

In the 10- and 20-min application group at the time of puncture of the arteriovenous fistula, both groups showed pain reduction, so the application time of thermotherapy was reduced to 10 min. In previous studies, the application time of thermotherapy was 20–30 min [3,12]. However, those studies required it to be applied differently, depending on the time and area, in order to increase the temperature of the tissue [26]. The result of the present study is similar to the study result that the time required to reach the maximum temperature when applying the hot pack to the lumbar region in the supine position is 10 min [27]. This was a result of scientifically reducing the application time when performing thermotherapy before puncture of the arteriovenous fistula. Moreover, the time required to apply thermotherapy before puncture of the arteriovenous fistula was too long, making it difficult to provide nursing interventions at busy clinical sites. The reduction of the application time for intervention is expected to help reduce the workload of nurses and provide active interventions.

The feeling of anxiety among patients in the 10- and 20-min thermotherapy groups decreased compared to the anxiety of the control group, but there was no statistically significant difference between the three groups. Thermotherapy is known to provide comfort [28], but in this study, thermotherapy did not affect anxiety reduction. This is the opposite of previous studies’ findings, which showed a reduction of anxiety after treatment when thermotherapy was applied during puncture of the arteriovenous fistula [29]. This was because the earlier studies were not designed as randomized controlled trials. 

Subjects with arteriovenous fistula feel anxiety more if they have previously experienced pain and if pain is expected in the future. Conversely, when they have anxiety or muscle tension, the pain increases [30]. Hemodialysis patients experience uncertainty, anxiety, and negative emotions, along with the pain associated with the disease treatment process [31]. They thereby form a negative self-concept and show a negative response to the surrounding environment [32]. Although the reduction of anxiety with thermotherapy has not been scientifically confirmed, nurses should recognize anxiety as a problem to be solved and provide scientifically proven nursing interventions.

In this study, four subjects in the group to which thermotherapy was applied for 20 min complained of pruritus as a side effect. Because of the differences in the skin tissue, skin temperature, skin thickness, skin color, gender, race, etc., even if intervention is applied according to scientifically confirmed procedures, nurses must carefully check the side effects of thermotherapy. Previous studies did not favor pharmacological intervention as it was considered to be an economic burden on the patient and also added to the pretreatment time [5,6,29]. The application of thermotherapy, which is a non-pharmacological intervention, maximizes the effectiveness of nursing interventions and can be applied effectively in the clinical setting. This study provides a scientific basis for providing safe and efficient nursing interventions to patients.

### Limitations

In this study, a single-blind approach was used so that researchers, intervention providers, and outcome measurers could not know about the interventions performed on the subjects. Full blinding was not applied since the subject was not aware of the assigned intervention. Therefore, in future studies, it will be necessary to study the placebo group to confirm the scientific effect of the intervention.

## 5. Conclusions

This study was a randomized controlled trial conducted to compare the application time of thermotherapy to reduce pain, anxiety, and adverse events during puncture of the arteriovenous fistula in hemodialysis patients. The study showed there was no difference in pain between 10- and 20-min thermotherapy groups at the time of puncture. However, the two experimental groups had reduced pain compared to the control group. In clinical practice, the application time of thermotherapy for pain reduction during arteriovenous fistula puncture has been applied in various ways, but the application time could be safely shortened.

## Figures and Tables

**Table 1:** General characteristics and homogeneity of the three groups.

Variables		10-min Group (*n* = 26)	20-min Group (*n* = 26)	Control Group (*n* = 25)	F or χ^2^	*p*
		*N*(%) or M ± SD	*N*(%) or M ± SD	*N*(%) or M ± SD		
Age (year)		60.2 ± 10.8	63.7 ± 8.9	56.7 ± 13.5	2.528	0.087
Sex	Male	15 (57.7)	14 (53.9)	17 (68.0)	1.130	0.568
	Female	11 (42.3)	12 (46.2)	8 (32.0)		
Diabetes Mlietus	Yes	12 (46.2)	16 (61.5)	8 (32.0)	4.473	0.107
	No	14 (53.8)	10 (38.5)	17 (68.0)		
Hypertension	Yes	5 (19.2)	7 (26.9)	8 (32.0)	1.099	0.577
	No	21 (80.8)	19 (73.1)	17 (68.0)		
Glomerulo-nephritis	Yes	1 (3.8)	2 (7.7)	4 (16.0)	2.371	0.306
	No	25 (96.2)	24 (92.3)	21 (84.0)		
Treatment period of hemodialysis (year)		38.9 ± 49.6	57.6 ± 63.6	66.3 ± 63.0	1.437	0.244
Cycle of hemodialysis	3 times per week	26 (100.0)	25 (96.1)	25 (100.0)	1.987	0.370
	2 times per week	0 (0)	1 (3.9)	0 (0)		
Type of arteriovenous fistula	Fistula	23 (88.5)	20 (76.9)	24 (96.0)	4.177	0.124
	Graft	3 (11.5)	6 (23.1)	1 (4.0)		
Site of arteriovenous fistula	Upper arm	15 (57.7)	11 (42.3)	11 (44.0)	1.748	0.782
	Forearm	9 (34.6)	11 (42.3)	11 (44.0)		
	Upper arm and forearm	2 (7.7)	4 (15.4)	3 (12.0)		
Use of lidocaine cream	Yes	0 (0)	0 (0)	0 (0)		
	No	26 (100)	26 (100)	25 (100)		
Pain before arteriovenous fistula puncture		4.4 ± 1.9	4.5 ± 1.9	4.3 ± 1.7	0.038	0.963
Anxiety before arteriovenous fistula puncture		4.0 ± 1.9	3.6 ± 1.9	3.2 ± 1.3	1.587	0.211

**Table 2:** Differences of pain and anxiety of three groups.

Variables	10-min Group ^a^ (*n* = 26)	20-min Group ^b^ (*n* = 26)	Control Group ^c^ (*n* = 25)	F	*p*
	Mean ± SD	Mean ± SD	Mean ± SD		
Pain	3.4 ± 1.8	3.2 ± 1.1	5.1 ± 1.6	11.457	<0.001
Anxiety	3.3 ± 1.8	3.0 ± 1.6	3.2 ± 1.4	0.255	0.775

a, b < c. a: 10-min Group; b: 20-min Group; c: Control Group

**Table 3:** Comparison of side effects for the three groups.

Side Effects		10-min Group (*n* = 26) *n* (%)	20-min Group (*n* = 26) *n* (%)	Control Group (*n* = 25) *n* (%)
Redness	Yes	0 (0)	0 (0)	0 (0)
	No	26 (100)	26 (100)	25 (100)
Itching	Yes	0 (0)	4 (15)	0 (0)
	No	26 (100)	22 (85)	25 (100)
Edema	Yes	0 (0)	0 (0)	0 (0)
	No	26 (100)	26 (100)	25 (100)
Bullous	Yes	0 (0)	0 (0)	0 (0)
	No	26 (100)	26 (100)	25 (100)
